# Ethyl 2-[4-(4-chloro­benzo­yl)phen­oxy]-2-methyl­propano­ate

**DOI:** 10.1107/S1600536812021149

**Published:** 2012-05-16

**Authors:** Zhao Yang, Zhi-Xiang Wang

**Affiliations:** a Department of Pharmaceutical Engineering, China Pharmaceutical University, Tongjiaxiang No. 24 Nanjing, Nanjing 210009, People’s Republic of China

## Abstract

In the title compound, C_19_H_19_ClO_4_, the dihedral angle between the mean planes of the benzene rings is 126.8 (1)°. Weak C—H⋯O inter­actions are observed.

## Related literature

For background, see: Guichard *et al.* (2000 ▶). For the synthesis of the title compound, see: Bandgar *et al.*, (2011 ▶). For reference bond-length data, see: Allen *et al.* (1987 ▶).
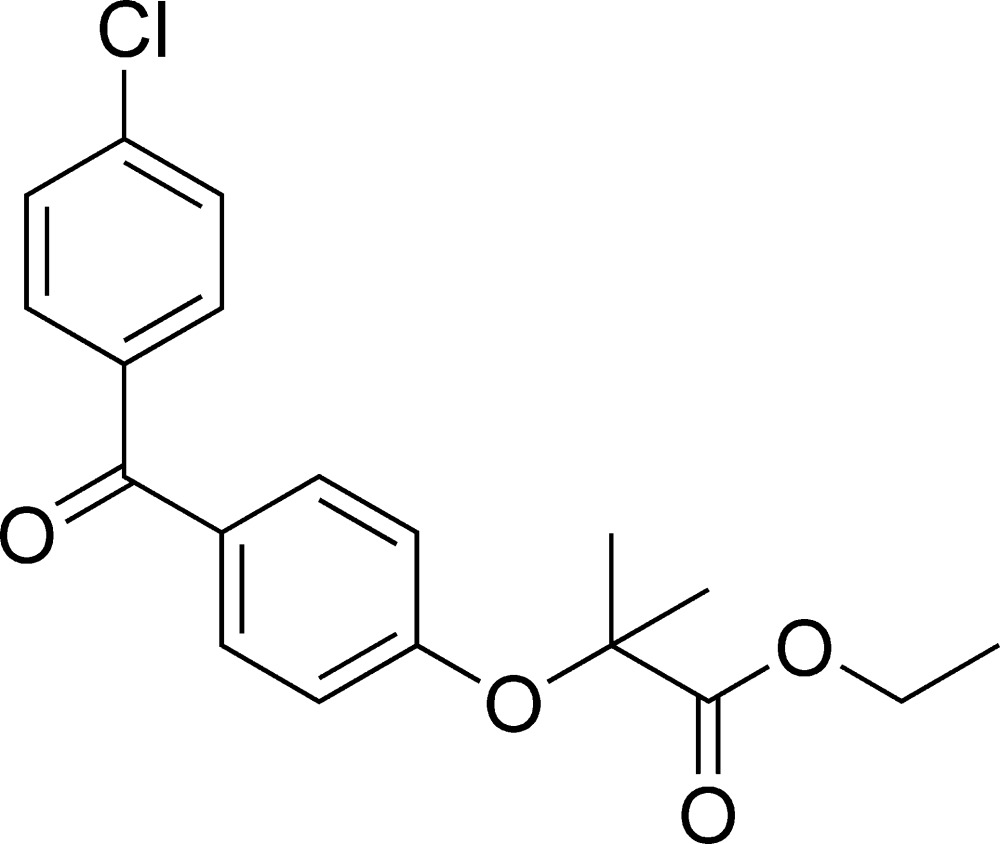


## Experimental

### 

#### Crystal data

C_19_H_19_ClO_4_
*M* *_r_* = 346.79
Orthorhombic, 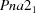

*a* = 13.677 (3) Å
*b* = 16.420 (3) Å
*c* = 7.9490 (16) Å
*V* = 1785.2 (6) Å^3^
*Z* = 4
Mo *K*α radiation
μ = 0.23 mm^−1^
*T* = 293 K
0.20 × 0.20 × 0.10 mm

#### Data collection

Enraf–Nonius CAD-4 diffractometer
Absorption correction: ψ scan (North *et al.*, 1968 ▶) *T*_min_ = 0.955, *T*_max_ = 0.977
3465 measured reflections
3459 independent reflections
1971 reflections with *I* > 2σ(*I*)
*R*_int_ = 0.058

#### Refinement

*R*[*F* ^2^ > 2σ(*F* ^2^)] = 0.068
*wR*(*F* ^2^) = 0.180
*S* = 1.00
3459 reflections
218 parameters
1 restraint
H-atom parameters constrained
Δρ_max_ = 0.21 e Å^−3^
Δρ_min_ = −0.21 e Å^−3^
Absolute structure: Flack (1983 ▶), with 1692 Friedel pairs
Flack parameter: 0.04 (17)

### 

Data collection: *CAD-4 EXPRESS* (Enraf–Nonius, 1989 ▶); cell refinement: *CAD-4 EXPRESS*; data reduction: *XCAD4* (Harms & Wocadlo, 1995 ▶); program(s) used to solve structure: *SHELXS97* (Sheldrick, 2008 ▶); program(s) used to refine structure: *SHELXL97* (Sheldrick, 2008 ▶); molecular graphics: *SHELXTL-Plus* (Sheldrick, 2008 ▶); software used to prepare material for publication: *SHELXL97*.

## Supplementary Material

Crystal structure: contains datablock(s) global, I. DOI: 10.1107/S1600536812021149/jj2137sup1.cif

Structure factors: contains datablock(s) I. DOI: 10.1107/S1600536812021149/jj2137Isup2.hkl

Supplementary material file. DOI: 10.1107/S1600536812021149/jj2137Isup3.cml

Additional supplementary materials:  crystallographic information; 3D view; checkCIF report

## Figures and Tables

**Table 1:** Hydrogen-bond geometry (Å, °)

*D*—H⋯*A*	*D*—H	H⋯*A*	*D*⋯*A*	*D*—H⋯*A*
C1—H1*A*⋯O3^i^	0.93	2.54	3.340 (7)	144

Symmetry code: (i) 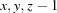.

**Fig. 1.**
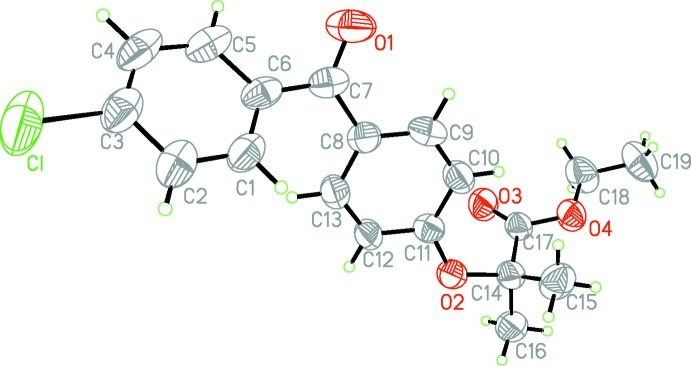
The molecular structure of the title compound, (I), showing the atom-labeling scheme and 50% probability displacement ellipsoids.

**Fig. 2.**
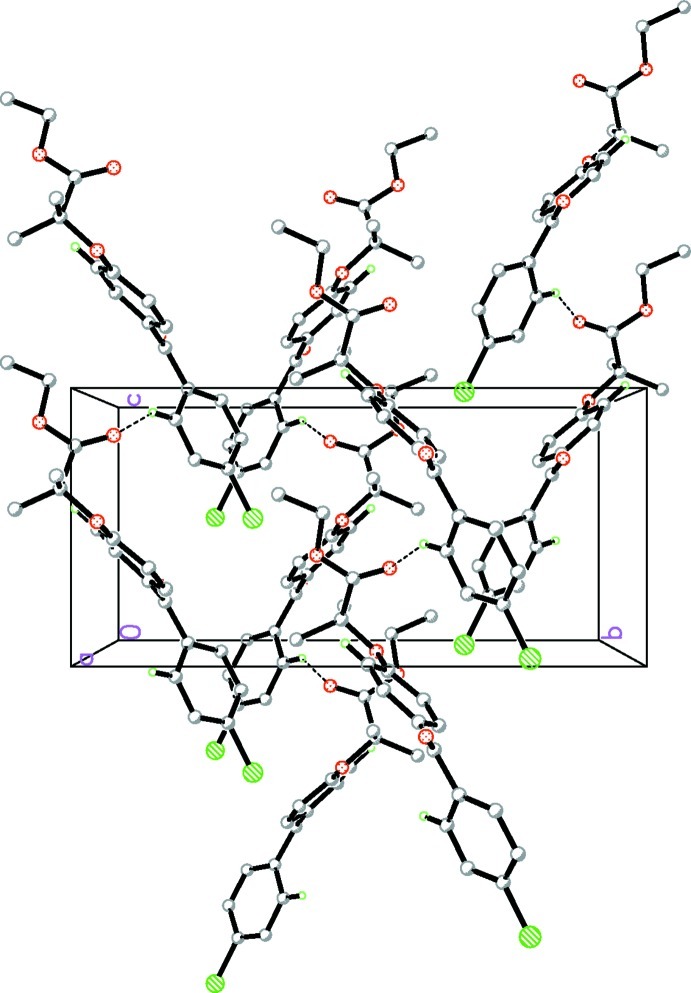
Packing diagram of the title compound viewed along the a axis. Dashed lines indicate weak C—H···O intermolecular interactions. Remaining H atoms have been omitted for clarity.

## supplementary crystallographic information

### Comment

The title compound, C_20_H_21_ClO_4_, (I), is Fenofibrate, an antihypertensive drug (Guichard *et al.* 2000). We report herein its crystal structure.

In the title compound, (I), the dihedral angle between the mean planes of the benzene and phenyl rings is 126.8 (1)°. Crystal packing is influenced by weak C—H···O intermolecular interactions. Bond lengths are in normal ranges (Allen *et al.* 1987).

### Experimental

2-(4-(4-chlorobenzoyl)phenoxy)-2-methylpropanoic acid (6.28 mmol, 2.00 g) was dissolved in 35% hydrochloric acid ethanol solution (15 ml), the solution was heated to 338.15 K under N_2_ atmosphere for 3 h. The reaction mexture was cooled to room temperature and the solvent was distilled to get the crude compound. The crude compond was dissolved in dichloromethane (15 ml), washed with water (10 ml) three times, dried, and concentrated to get the title compound (1.95 g). pure: white solid (Bandgar *et al.* 2011). Crystals of the title compound suitable for X-ray diffraction were obtained by slow evaporation of an ethanol solution.

### Refinement

H atoms were positioned geometrically with C—H = 0.93, 0.98 and 0.97 Å for aromatic, methine and methylene H atoms, respectively, and constrained to ride on their parent atoms, with *U*_iso_(H) = 1.2 (or 1.5 for methyl groups) times *U*_eq_(C). 1692 Friedel pairs were measured.

### Crystal data

**Table:**

C_19_H_19_ClO_4_	*F*(000) = 728
*M**_r_* = 346.79	*D*_x_ = 1.290 Mg m^−^^3^
Orthorhombic, *P**n**a*2_1_	Mo *K*α radiation, λ = 0.71073 Å
Hall symbol: P 2c -2n	Cell parameters from 25 reflections
*a* = 13.677 (3) Å	θ = 9–12°
*b* = 16.420 (3) Å	µ = 0.23 mm^−^^1^
*c* = 7.9490 (16) Å	*T* = 293 K
*V* = 1785.2 (6) Å^3^	Block, colourless
*Z* = 4	0.20 × 0.20 × 0.10 mm

### Data collection

**Table:**

Enraf–Nonius CAD-4 diffractometer	3459 independent reflections
Radiation source: fine-focus sealed tube	1971 reflections with *I* > 2σ(*I*)
Graphite monochromator	*R*_int_ = 0.058
Detector resolution: 16.0355 pixels mm^-1^	θ_max_ = 25.4°, θ_min_ = 2.5°
ω/2θ scans	*h* = 0→16
Absorption correction: ψ scan (North *et al.*, 1968)	*k* = −19→19
*T*_min_ = 0.955, *T*_max_ = 0.977	*l* = 0→9
3465 measured reflections	

### Refinement

**Table:**

Refinement on *F*^2^	Hydrogen site location: inferred from neighbouring sites
Least-squares matrix: full	H-atom parameters constrained
*R*[*F*^2^ > 2σ(*F*^2^)] = 0.068	*w* = 1/[σ^2^(*F*_o_^2^) + (0.078*P*)^2^] where *P* = (*F*_o_^2^ + 2*F*_c_^2^)/3
*wR*(*F*^2^) = 0.180	(Δ/σ)_max_ < 0.001
*S* = 1.00	Δρ_max_ = 0.21 e Å^−^^3^
3459 reflections	Δρ_min_ = −0.21 e Å^−^^3^
218 parameters	Extinction correction: *SHELXL97* (Sheldrick, 2008), Fc^*^=kFc[1+0.001xFc^2^λ^3^/sin(2θ)]^-1/4^
1 restraint	Extinction coefficient: 0.029 (3)
Primary atom site location: structure-invariant direct methods	Absolute structure: Flack (1983), with 1692 Friedel pairs
Secondary atom site location: difference Fourier map	Flack parameter: 0.04 (17)

### Special details

**Table:**

Geometry. All esds (except the esd in the dihedral angle between two l.s. planes) are estimated using the full covariance matrix. The cell esds are taken into account individually in the estimation of esds in distances, angles and torsion angles; correlations between esds in cell parameters are only used when they are defined by crystal symmetry. An approximate (isotropic) treatment of cell esds is used for estimating esds involving l.s. planes.
Refinement. Refinement of F^2^ against ALL reflections. The weighted R-factor wR and goodness of fit S are based on F^2^, conventional R-factors R are based on F, with F set to zero for negative F^2^. The threshold expression of F^2^ > 2sigma(F^2^) is used only for calculating R-factors(gt) etc. and is not relevant to the choice of reflections for refinement. R-factors based on F^2^ are statistically about twice as large as those based on F, and R-factors based on ALL data will be even larger.

### Fractional atomic coordinates and isotropic or equivalent isotropic displacement parameters (Å^2^)

**Table:**

	*x*	*y*	*z*	*U*_iso_*/*U*_eq_	
Cl	0.48890 (13)	0.70175 (10)	0.0275 (3)	0.1012 (7)	
O1	0.5732 (3)	0.8850 (3)	0.7608 (6)	0.0837 (13)	
C1	0.4206 (4)	0.8448 (3)	0.4133 (8)	0.0591 (15)	
H1A	0.3735	0.8840	0.4365	0.071*	
O2	0.1445 (2)	0.96725 (19)	1.0184 (5)	0.0586 (10)	
C2	0.4204 (4)	0.8080 (3)	0.2568 (8)	0.0679 (17)	
H2A	0.3747	0.8228	0.1757	0.081*	
O3	0.2113 (3)	0.9435 (2)	1.3404 (6)	0.0726 (13)	
C3	0.4895 (4)	0.7489 (3)	0.2235 (9)	0.0654 (17)	
O4	0.2075 (3)	1.0773 (2)	1.3897 (5)	0.0620 (11)	
C4	0.5574 (4)	0.7267 (3)	0.3426 (9)	0.0690 (19)	
H4A	0.6030	0.6861	0.3196	0.083*	
C5	0.5571 (4)	0.7653 (3)	0.4965 (9)	0.0629 (16)	
H5A	0.6041	0.7510	0.5757	0.075*	
C6	0.4886 (3)	0.8254 (3)	0.5378 (9)	0.0513 (13)	
C7	0.4929 (3)	0.8691 (3)	0.7002 (7)	0.0536 (14)	
C8	0.4017 (4)	0.8941 (3)	0.7865 (7)	0.0520 (14)	
C9	0.4053 (4)	0.9562 (3)	0.9038 (8)	0.0574 (15)	
H9A	0.4650	0.9809	0.9267	0.069*	
C10	0.3229 (4)	0.9827 (3)	0.9882 (7)	0.0573 (14)	
H10A	0.3272	1.0245	1.0668	0.069*	
C11	0.2338 (4)	0.9463 (3)	0.9542 (7)	0.0479 (13)	
C12	0.2301 (4)	0.8814 (3)	0.8440 (7)	0.0541 (14)	
H12A	0.1711	0.8545	0.8270	0.065*	
C13	0.3114 (4)	0.8558 (3)	0.7596 (8)	0.0580 (15)	
H13A	0.3069	0.8128	0.6839	0.070*	
C14	0.1328 (4)	1.0324 (3)	1.1404 (7)	0.0509 (13)	
C15	0.1562 (4)	1.1156 (3)	1.0644 (7)	0.0706 (17)	
H15A	0.2247	1.1185	1.0390	0.106*	
H15B	0.1395	1.1575	1.1434	0.106*	
H15C	0.1191	1.1229	0.9630	0.106*	
C16	0.0242 (4)	1.0277 (3)	1.1840 (8)	0.0731 (18)	
H16A	0.0101	0.9757	1.2338	0.110*	
H16B	−0.0139	1.0342	1.0834	0.110*	
H16C	0.0081	1.0702	1.2621	0.110*	
C17	0.1892 (3)	1.0110 (3)	1.3001 (7)	0.0512 (13)	
C18	0.2573 (5)	1.0655 (4)	1.5498 (9)	0.0796 (19)	
H18A	0.3151	1.0322	1.5333	0.096*	
H18B	0.2144	1.0374	1.6278	0.096*	
C19	0.2854 (5)	1.1450 (4)	1.6197 (10)	0.098 (2)	
H19A	0.3193	1.1372	1.7241	0.148*	
H19B	0.2278	1.1771	1.6389	0.148*	
H19C	0.3274	1.1727	1.5416	0.148*	

### Atomic displacement parameters (Å^2^)

**Table:**

	*U*^11^	*U*^22^	*U*^33^	*U*^12^	*U*^13^	*U*^23^
Cl	0.1114 (14)	0.0824 (11)	0.1098 (14)	0.0005 (9)	0.0354 (14)	−0.0362 (12)
O1	0.045 (2)	0.118 (3)	0.088 (3)	−0.003 (2)	−0.003 (2)	−0.001 (3)
C1	0.054 (3)	0.048 (3)	0.075 (4)	0.009 (3)	0.010 (3)	−0.003 (3)
O2	0.045 (2)	0.072 (2)	0.059 (2)	0.0055 (16)	−0.002 (2)	−0.022 (2)
C2	0.073 (4)	0.055 (3)	0.076 (4)	0.008 (3)	0.015 (4)	−0.004 (3)
O3	0.084 (3)	0.049 (2)	0.085 (3)	0.002 (2)	−0.023 (2)	0.003 (2)
C3	0.059 (4)	0.053 (3)	0.084 (4)	0.000 (3)	0.025 (4)	−0.004 (3)
O4	0.073 (3)	0.060 (2)	0.053 (2)	0.002 (2)	−0.013 (2)	−0.009 (2)
C4	0.052 (3)	0.049 (3)	0.106 (6)	0.007 (3)	0.034 (4)	0.005 (4)
C5	0.049 (3)	0.057 (3)	0.082 (5)	0.013 (3)	0.018 (4)	0.020 (4)
C6	0.038 (3)	0.048 (3)	0.068 (4)	0.001 (2)	0.014 (3)	0.015 (3)
C7	0.040 (3)	0.060 (3)	0.061 (4)	0.003 (2)	0.003 (3)	0.012 (3)
C8	0.045 (3)	0.052 (3)	0.059 (4)	0.002 (2)	−0.002 (3)	0.000 (3)
C9	0.040 (3)	0.068 (3)	0.064 (4)	−0.009 (3)	−0.006 (3)	0.007 (3)
C10	0.055 (3)	0.062 (3)	0.055 (4)	−0.007 (3)	−0.003 (3)	−0.012 (3)
C11	0.044 (3)	0.055 (3)	0.044 (3)	−0.001 (2)	−0.001 (3)	0.000 (3)
C12	0.041 (3)	0.057 (3)	0.065 (4)	−0.007 (2)	0.003 (3)	−0.020 (3)
C13	0.052 (3)	0.056 (3)	0.066 (4)	−0.007 (2)	0.002 (3)	−0.019 (3)
C14	0.049 (3)	0.055 (3)	0.048 (3)	0.001 (2)	−0.003 (3)	−0.008 (3)
C15	0.084 (4)	0.065 (3)	0.063 (4)	0.015 (3)	−0.010 (3)	0.007 (3)
C16	0.054 (4)	0.085 (4)	0.080 (5)	0.012 (3)	0.000 (3)	−0.027 (4)
C17	0.048 (3)	0.057 (3)	0.048 (3)	0.000 (3)	−0.002 (3)	−0.011 (3)
C18	0.103 (5)	0.078 (4)	0.058 (4)	−0.012 (4)	−0.029 (4)	0.004 (4)
C19	0.105 (5)	0.103 (5)	0.088 (5)	−0.029 (4)	−0.012 (5)	−0.024 (5)

### Geometric parameters (Å, º)

**Table:**

Cl—C3	1.740 (7)	C9—H9A	0.9300
O1—C7	1.227 (6)	C10—C11	1.383 (7)
C1—C2	1.383 (8)	C10—H10A	0.9300
C1—C6	1.395 (7)	C11—C12	1.380 (7)
C1—H1A	0.9300	C12—C13	1.365 (7)
O2—C11	1.369 (6)	C12—H12A	0.9300
O2—C14	1.452 (6)	C13—H13A	0.9300
C2—C3	1.380 (7)	C14—C17	1.527 (7)
C2—H2A	0.9300	C14—C15	1.527 (7)
O3—C17	1.192 (6)	C14—C16	1.527 (7)
C3—C4	1.376 (8)	C15—H15A	0.9600
O4—C17	1.325 (6)	C15—H15B	0.9600
O4—C18	1.457 (7)	C15—H15C	0.9600
C4—C5	1.378 (8)	C16—H16A	0.9600
C4—H4A	0.9300	C16—H16B	0.9600
C5—C6	1.399 (7)	C16—H16C	0.9600
C5—H5A	0.9300	C18—C19	1.470 (7)
C6—C7	1.478 (8)	C18—H18A	0.9700
C7—C8	1.482 (7)	C18—H18B	0.9700
C8—C9	1.382 (7)	C19—H19A	0.9600
C8—C13	1.401 (7)	C19—H19B	0.9600
C9—C10	1.382 (7)	C19—H19C	0.9600
			
C2—C1—C6	122.7 (5)	C11—C12—H12A	119.4
C2—C1—H1A	118.7	C12—C13—C8	120.3 (5)
C6—C1—H1A	118.7	C12—C13—H13A	119.8
C11—O2—C14	122.2 (4)	C8—C13—H13A	119.8
C3—C2—C1	118.5 (6)	O2—C14—C17	109.3 (4)
C3—C2—H2A	120.7	O2—C14—C15	111.8 (5)
C1—C2—H2A	120.7	C17—C14—C15	115.4 (4)
C4—C3—C2	121.2 (6)	O2—C14—C16	102.8 (4)
C4—C3—Cl	120.1 (4)	C17—C14—C16	106.9 (5)
C2—C3—Cl	118.8 (5)	C15—C14—C16	109.8 (4)
C17—O4—C18	116.6 (4)	C14—C15—H15A	109.5
C3—C4—C5	119.1 (5)	C14—C15—H15B	109.5
C3—C4—H4A	120.5	H15A—C15—H15B	109.5
C5—C4—H4A	120.5	C14—C15—H15C	109.5
C4—C5—C6	122.4 (6)	H15A—C15—H15C	109.5
C4—C5—H5A	118.8	H15B—C15—H15C	109.5
C6—C5—H5A	118.8	C14—C16—H16A	109.5
C1—C6—C5	116.2 (6)	C14—C16—H16B	109.5
C1—C6—C7	122.4 (4)	H16A—C16—H16B	109.5
C5—C6—C7	121.4 (5)	C14—C16—H16C	109.5
O1—C7—C6	118.8 (5)	H16A—C16—H16C	109.5
O1—C7—C8	120.8 (5)	H16B—C16—H16C	109.5
C6—C7—C8	120.3 (5)	O3—C17—O4	124.9 (5)
C9—C8—C13	117.7 (5)	O3—C17—C14	124.4 (5)
C9—C8—C7	119.1 (5)	O4—C17—C14	110.7 (4)
C13—C8—C7	123.1 (5)	O4—C18—C19	109.5 (5)
C10—C9—C8	122.0 (5)	O4—C18—H18A	109.8
C10—C9—H9A	119.0	C19—C18—H18A	109.8
C8—C9—H9A	119.0	O4—C18—H18B	109.8
C9—C10—C11	119.2 (5)	C19—C18—H18B	109.8
C9—C10—H10A	120.4	H18A—C18—H18B	108.2
C11—C10—H10A	120.4	C18—C19—H19A	109.5
O2—C11—C12	113.4 (4)	C18—C19—H19B	109.5
O2—C11—C10	127.2 (5)	H19A—C19—H19B	109.5
C12—C11—C10	119.3 (5)	C18—C19—H19C	109.5
C13—C12—C11	121.3 (5)	H19A—C19—H19C	109.5
C13—C12—H12A	119.4	H19B—C19—H19C	109.5
			
C6—C1—C2—C3	0.9 (8)	C14—O2—C11—C12	178.2 (5)
C1—C2—C3—C4	0.0 (8)	C14—O2—C11—C10	−1.9 (8)
C1—C2—C3—Cl	179.4 (4)	C9—C10—C11—O2	−176.3 (5)
C2—C3—C4—C5	−1.2 (8)	C9—C10—C11—C12	3.6 (8)
Cl—C3—C4—C5	179.5 (4)	O2—C11—C12—C13	175.6 (5)
C3—C4—C5—C6	1.5 (8)	C10—C11—C12—C13	−4.4 (8)
C2—C1—C6—C5	−0.6 (7)	C11—C12—C13—C8	1.6 (9)
C2—C1—C6—C7	175.4 (5)	C9—C8—C13—C12	1.8 (8)
C4—C5—C6—C1	−0.6 (7)	C7—C8—C13—C12	179.5 (5)
C4—C5—C6—C7	−176.7 (5)	C11—O2—C14—C17	−62.1 (6)
C1—C6—C7—O1	−140.2 (5)	C11—O2—C14—C15	66.9 (6)
C5—C6—C7—O1	35.6 (7)	C11—O2—C14—C16	−175.4 (5)
C1—C6—C7—C8	39.0 (7)	C18—O4—C17—O3	−0.7 (8)
C5—C6—C7—C8	−145.2 (5)	C18—O4—C17—C14	177.4 (5)
O1—C7—C8—C9	20.4 (7)	O2—C14—C17—O3	−22.5 (7)
C6—C7—C8—C9	−158.8 (5)	C15—C14—C17—O3	−149.5 (5)
O1—C7—C8—C13	−157.2 (5)	C16—C14—C17—O3	88.1 (6)
C6—C7—C8—C13	23.5 (7)	O2—C14—C17—O4	159.4 (4)
C13—C8—C9—C10	−2.5 (8)	C15—C14—C17—O4	32.4 (6)
C7—C8—C9—C10	179.7 (5)	C16—C14—C17—O4	−90.0 (5)
C8—C9—C10—C11	−0.2 (8)	C17—O4—C18—C19	171.2 (5)

### Hydrogen-bond geometry (Å, º)

**Table:**

*D*—H···*A*	*D*—H	H···*A*	*D*···*A*	*D*—H···*A*
C1—H1*A*···O3^i^	0.93	2.54	3.340 (7)	144

Symmetry code: (i) *x*, *y*, *z*−1.
